# Public awareness of early symptoms of acute coronary syndrome and its association with anticipated prehospital delay: a cross-sectional study in Palestine

**DOI:** 10.3389/fpubh.2026.1856753

**Published:** 2026-07-23

**Authors:** Musab Basem Dwaik, Khaled Alhashlamon, Haya Mustafa, Mahmoud Al-Swaiti, Ahmad Abu Kbash, Lana R. Qasem, Dalal Albada, Amira Saqer, Lena M. S. Abulkhair, Salahaldeen Deeb

**Affiliations:** 1 Faculty of Medicine, Al-Quds University, Jerusalem, Palestine; 2 Faculty of Medicine, Al-Najah University, Nablus, Palestine; 3 Faculty of Medicine, Hebron University, Hebron, Palestine

**Keywords:** acute coronary syndrome, cardiovascular risk, emergency response, health behavior, Palestine, prehospital delay, symptom awareness

## Abstract

**Background:**

Timely recognition of acute coronary syndrome (ACS) symptoms is critical for early intervention and improved outcomes. This study assessed public awareness of ACS symptoms and its association with anticipated prehospital delay in Palestine.

**Methods:**

A cross-sectional online survey was conducted between February and March 2026 among 1,293 Palestinian adults recruited through convenience sampling on social media platforms. A structured questionnaire assessed ACS symptom and risk-factor knowledge, anticipated help-seeking response to suspected ACS symptoms, and self-reported cardiovascular risk burden. Composite scores were pre-specified, and multivariable regression models were used to examine factors associated with knowledge and anticipated delay risk.

**Results:**

The mean knowledge score was 7.91 ± 2.96, with 33.1% demonstrating high knowledge. A marked knowledge-action gap was observed: although 90.1% identified ambulance activation as the correct action, only 56.2% reported that they would call an ambulance for chest pain lasting more than 5 min. Higher knowledge was weakly associated with a more appropriate anticipated response (*β* = 0.083, *p* < 0.001), whereas higher cardiovascular risk burden was associated with poorer anticipated response (OR = 1.22, 95% CI 1.01–1.48). Females had higher knowledge scores, while males reported more appropriate anticipated emergency responses.

**Conclusion:**

This study identified suboptimal ACS knowledge and a gap between awareness and intended emergency response in an online convenience sample of Palestinian adults. Because the behavioral outcome was hypothetical, findings should be interpreted as anticipated response rather than actual prehospital delay. Interventions should combine symptom-awareness education with behavior-change strategies that promote immediate emergency medical service activation.

## Introduction

Acute coronary syndrome (ACS), encompassing ST-segment elevation myocardial infarction (STEMI), non-ST-segment elevation myocardial infarction (NSTEMI), and unstable angina, remains a leading cause of morbidity and mortality worldwide (1, 2). Despite substantial advances in diagnostic modalities and reperfusion therapies, outcomes in ACS are highly dependent on early recognition and timely initiation of treatment (2, 3).

Prehospital delay, defined as the time from symptom onset to first medical contact, is a critical determinant of clinical outcomes. Prolonged delay is associated with increased myocardial damage and mortality. Importantly, most of this delay is patient-related, often resulting from poor symptom recognition and misinterpretation of symptom severity (3, 4). Although early reperfusion is essential, only a minority of patients present within the recommended time window, while many delay seeking care for several hours (4).

Public awareness and recognition of early ACS symptoms play a crucial role in timely help-seeking behavior. While chest pain is the most commonly recognized symptom, ACS may also present with atypical manifestations such as dyspnea, fatigue, nausea, or indigestion, which are frequently misattributed to less serious conditions (5, 6). Consequently, patients experiencing atypical or gradual symptom onset are more likely to delay seeking medical attention. In addition, psychological factors, including denial, low perceived risk, and symptom misinterpretation, further contribute to delayed decision-making (6, 7).

Previous studies have demonstrated that a substantial proportion of the population is unable to identify key ACS symptoms or respond appropriately in emergency situations (5, 7). Limited knowledge of ACS symptoms has been consistently associated with longer prehospital delay, regardless of a country’s income level (7, 8). Furthermore, sociodemographic factors, including age, sex, education level, and socioeconomic status, significantly influence symptom recognition and healthcare-seeking behavior (8, 9). Higher levels of education and prior knowledge of cardiovascular disease are associated with improved symptom recognition and shorter delays in seeking care (8, 9).

Evidence from patient-based studies supports a direct link between symptom knowledge, symptom attribution, and prehospital delay. In a study of first acute myocardial infarction patients in Pakistan, lack of knowledge of heart attack symptoms independently increased the odds of delayed presentation, and many participants failed to recognize their symptoms as myocardial infarction. Similarly, research among ACS survivors found that better knowledge of cardiovascular risk factors, better ACS symptom knowledge, and attribution of symptoms to a cardiac cause were associated with shorter patient decision delay. These findings indicate that poor symptom recognition and misattribution are not only awareness problems but also behavioral barriers to timely care (10, 11).

Cardiovascular disease represents a major health burden in the Middle East, including Palestine. However, data on public awareness of ACS symptoms and anticipated responses to symptom onset in this context remain limited. Existing regional studies suggest that inadequate knowledge, cultural beliefs, and barriers to healthcare access contribute to delayed presentation and suboptimal outcomes (1, 5, 7, 12, 13). Variations in awareness across different demographic groups further highlight the need for targeted public health interventions in the region.

While numerous studies have examined factors associated with actual prehospital delay, fewer have explored anticipated or intended responses to hypothetical symptom scenarios. Understanding anticipated delay is important, as it reflects underlying knowledge and behavioral intentions. Emerging evidence suggests that anticipated responses may serve as a proxy for real-world behavior in ACS situations (14).

Therefore, this study aims to assess public awareness of early ACS symptoms and examine its association with anticipated prehospital delay among individuals in Palestine. Identifying gaps in knowledge and intended responses may inform the development of targeted educational strategies and public health interventions to reduce delays and improve cardiovascular outcomes.

## Methodology

### Study design and setting

A cross-sectional study was conducted using an online questionnaire among adults aged ≥ 18 years in Palestine. A total of 1,293 participants were included in the final analysis.

### Participants and eligibility criteria

The target population was Palestinian adults aged 18 years and older residing in Palestine. Recruitment was open to adults from different social and occupational backgrounds, including students, employed individuals, healthcare and non-healthcare professionals, homemakers, and older adults. Because recruitment was conducted online using convenience sampling, the sample was not designed to be nationally representative. Representativeness was assessed descriptively by comparing the sample distribution by age, sex, and education with available Palestinian Central Bureau of Statistics data.

The inclusion and exclusion criteria were adapted from (7) and contained the following:

- Inclusion criteria:

Participants aged 18 years old and above.

Participants are able to speak and understand Arabic.

Participants are conscious and oriented.

Participants have Palestinian nationality and are currently residing in Palestine.

Participants willing to participate in the research.

- Exclusion criteria:

Participants with any mental or psychological instability.

Participants below 18 years old.

Palestinian participants aren’t residing in Palestine.

Participants who have no online platforms.

Participants who refuse to participate in the research.

Participants with prior diagnoses of acute coronary syndrome (ACS) due to their personal experience with the disease.

### Sampling strategy and recruitment

A convenience sampling approach was employed using online platforms, including WhatsApp, Facebook, and Instagram, to recruit participants. This method was selected to facilitate data collection in light of contextual constraints in Palestine, including accessibility and mobility limitations.

This recruitment approach may have preferentially reached younger, more educated, internet-connected, and social-media-active adults. No probability-based sampling frame or population weighting was used; therefore, prevalence estimates should be interpreted as estimates within the recruited online sample rather than national population estimates.

Eligible individuals received an electronic invitation containing a brief description of the study objectives, eligibility criteria, and assurances of confidentiality. Participation was voluntary, and informed consent was obtained electronically prior to data collection.

Recruitment was conducted over the study period until the desired sample size was achieved, resulting in a final sample of 1,293 participants.

### Sample size determination

The minimum required sample size for this cross-sectional study was calculated using the standard formula for estimating a single population proportion. Assuming a 95% confidence level (*Z* = 1.96), a margin of error of 5%, and an expected proportion (*p*) of 50% to ensure maximum sample size in the absence of prior local estimates, the calculated minimum sample size was 384 participants.

To account for potential non-response and incomplete questionnaires, the sample size was increased by approximately 10–20%, resulting in a target sample size of at least 420 participants. Ultimately, a total of 1,293 participants were included in the analysis.

### Instrument development and content

The questionnaire was constructed through a staged process. First, the study team identified domains relevant to ACS symptom awareness, appropriate emergency response, anticipated delay, and cardiovascular risk burden from established population health instruments and prior ACS awareness studies. Cardiovascular risk-factor items were informed by the World Health Organization STEPwise approach to chronic disease risk-factor surveillance, while symptom-awareness and emergency-response items were informed by prior heart attack and ACS awareness surveys, including CDC-style heart attack symptom and emergency-response items. Items were then adapted to the Palestinian context, including local emergency medical service terminology and locally relevant barriers to seeking care (15).

The final questionnaire included four sections: sociodemographic characteristics; ACS symptom, risk-factor, and emergency-response knowledge; anticipated help-seeking behavior in response to suspected ACS symptoms; and self-reported cardiovascular risk factors and lifestyle behaviors. The ACS knowledge section included 13 scored items, the anticipated prehospital delay score included items on intended initial action, time to seek help, response under uncertainty, and EMS preparedness, and the cardiovascular risk burden index included seven self-reported risk factors.

Content validity was assessed by medical professionals who reviewed each item for relevance, clarity, and cultural appropriateness. The questionnaire was developed in English and translated into Arabic using forward-backward translation. Formal pilot testing and factor analysis were not performed before data collection; this is acknowledged as a limitation.

To ensure absolute methodological transparency and full reproducibility, the exact operational wording of the questionnaire items, item-level coding schema, and explicit step-by-step scoring algorithms utilized to construct the continuous composite indices and categorical classifications (ACS Knowledge Score, Anticipated Prehospital Delay Score, and Cardiovascular Risk Burden Index) have been comprehensively detailed in the Supplementary material (Appendix 1), allowing for future independent validation.

### Outcome definitions and scoring (pre-specified)

Three main outcome measures were pre-specified for analysis: knowledge score, anticipated prehospital delay score, and cardiovascular risk burden index.

The ACS knowledge score was calculated from 13 pre-specified items assessing recognition of ACS symptoms, cardiovascular risk factors, and the correct immediate emergency action. Each correct response was assigned one point, while incorrect and “do not know” responses were scored as zero; total scores ranged from 0 to 13, with higher scores indicating greater knowledge. For descriptive categorical analyses, scores were grouped into low (0–5), moderate (6–9), and high (10–13) knowledge. These categories were defined *a priori* to approximate low, intermediate, and high performance across the possible score range and were not intended to represent externally validated clinical thresholds.

The anticipated prehospital delay score was derived from items assessing intended behavior in a suspected ACS scenario, including initial action, time to seek medical help, likelihood of delaying when symptom severity was uncertain, and EMS preparedness. Scores ranged from 0 to 8, with higher scores indicating more appropriate anticipated emergency response. For categorical analyses, scores were grouped into high delay risk (0–2), moderate delay risk (3–5), and low delay risk (6–8). These categories were used to aid interpretation; the continuous score was retained for correlation and linear regression analyses.

The cardiovascular risk burden index was calculated by summing seven self-reported risk factors: smoking, hypertension, diabetes, dyslipidemia, family history of cardiovascular disease, physical inactivity, and poor dietary habits. Each factor contributed one point, resulting in a total score from 0 to 7. Risk burden was categorized as low (0–2), moderate (3–4), and high (5–7). Because this index represents a formative count of different clinical and lifestyle risk factors rather than a reflective psychometric scale, internal consistency reliability was not considered conceptually appropriate for this index.

All outcome measures and score categories were defined prior to data analysis to ensure methodological consistency and reduce bias.

### Data collection procedures and quality control

The survey was hosted on Google Forms. To enhance data quality, required fields were set for key items, and submissions with <80% completion were excluded per protocol. The dataset was screened for obvious duplicate entries (e.g., repeated submissions with identical demographic profiles and near-identical response patterns/time stamps), which were removed before analysis. No personally identifying information was collected.

### Data management and handling of missing data

Data were exported to SPSS v27 for analysis. Records failing eligibility checks, suspected duplicates, and <80% completion were removed. For retained records, missing responses on awareness/knowledge items were coded as incorrect (0) in accordance with the scoring rubric; missingness patterns were examined descriptively.

### Statistical analysis

Statistical analyses were performed using SPSS version 27. Categorical variables were summarized as frequencies and percentages. Continuous composite scores were summarized using means and standard deviations and, where appropriate, medians and interquartile ranges. Distributional assumptions were assessed using histograms, Q–Q plots, and normality tests. Bivariate associations were examined using chi-square tests for categorical variables, *t*-tests or ANOVA for normally distributed continuous outcomes, non-parametric alternatives when assumptions were not met, and Spearman correlation for associations between ordinal or non-normally distributed scores.

Internal consistency reliability was assessed using Cronbach’s alpha for the ACS knowledge scale. The anticipated prehospital delay score and cardiovascular risk burden index were treated as composite indices of heterogeneous components rather than reflective psychometric scales; therefore, internal consistency was not calculated for these indices. Composite score categories were used for descriptive analyses, while continuous scores were retained in regression analyses when appropriate to reduce information loss.

Multivariable linear regression was used to identify factors associated with the continuous ACS knowledge score and the continuous anticipated prehospital delay score. Binary logistic regression was used to identify predictors of high anticipated delay risk, coded as 1 = high delay risk and 0 = moderate/low delay risk. Candidate predictors were selected based on prior literature, conceptual relevance, and bivariate associations, and included age, gender, education, health insurance status, healthcare worker status, ACS knowledge score, and cardiovascular risk burden where applicable. Regression assumptions were not fully available in the analysis output used for this revision; this limitation is acknowledged in the Discussion.

For logistic regression, odds ratios, 95% confidence intervals, and *p*-values were reported for all predictors available in the model output. Statistical significance was set at two-sided *p* < 0.05. A sensitivity analysis excluding healthcare workers was not available in the analysis output used for this revision and is therefore acknowledged as a limitation.

### Ethical consideration

This study was conducted in accordance with the principles of the Declaration of Helsinki. Ethical approval was obtained from the Research Ethics Committee at the authors’ institution (Ref No: 693/REC/2026).

Participation in the study was voluntary, and informed consent was obtained electronically from all participants prior to completing the questionnaire. Participants were informed about the purpose of the study, the anonymity of their responses, and their right to withdraw at any time without any consequences.

No personally identifiable information was collected, and all data were handled confidentially and used solely for research purposes.

## Results

### Participant characteristics and composite scores

A total of 1,293 participants were included in the final analysis. Baseline characteristics are presented in Table 1.

**Table 1:** Baseline sample characteristics.

Variable	Category	*n*	%
Age group	18–29 years	726	56.1
	30–39 years	265	20.5
	40–49 years	198	15.3
	50–59 years	77	6.0
	60 years and over	27	2.1
Gender	Female	786	60.8
	Male	507	39.2
Marital status	Single	688	53.2
	Married	570	44.1
	Divorced	26	2.0
	Widowed	9	0.7
Educational level	Bachelor’s	784	60.6
	Secondary	222	17.2
	Postgraduate	121	9.4
	Diploma	113	8.7
	Preparatory	43	3.3
	Primary or less	10	0.8
Residential area	City	679	52.5
	Village	516	39.9
	Camp	98	7.6
Health insurance	Yes	875	67.7
	No	418	32.3

The mean knowledge score was 7.91 ± 2.96 (median 8, IQR 6–10), the mean anticipated prehospital delay score was 5.91 ± 1.84 (median 6, IQR 5–7), and the mean cardiovascular risk burden index was 1.75 ± 1.29 (median 2, IQR 1–2).

**Figure 1:**
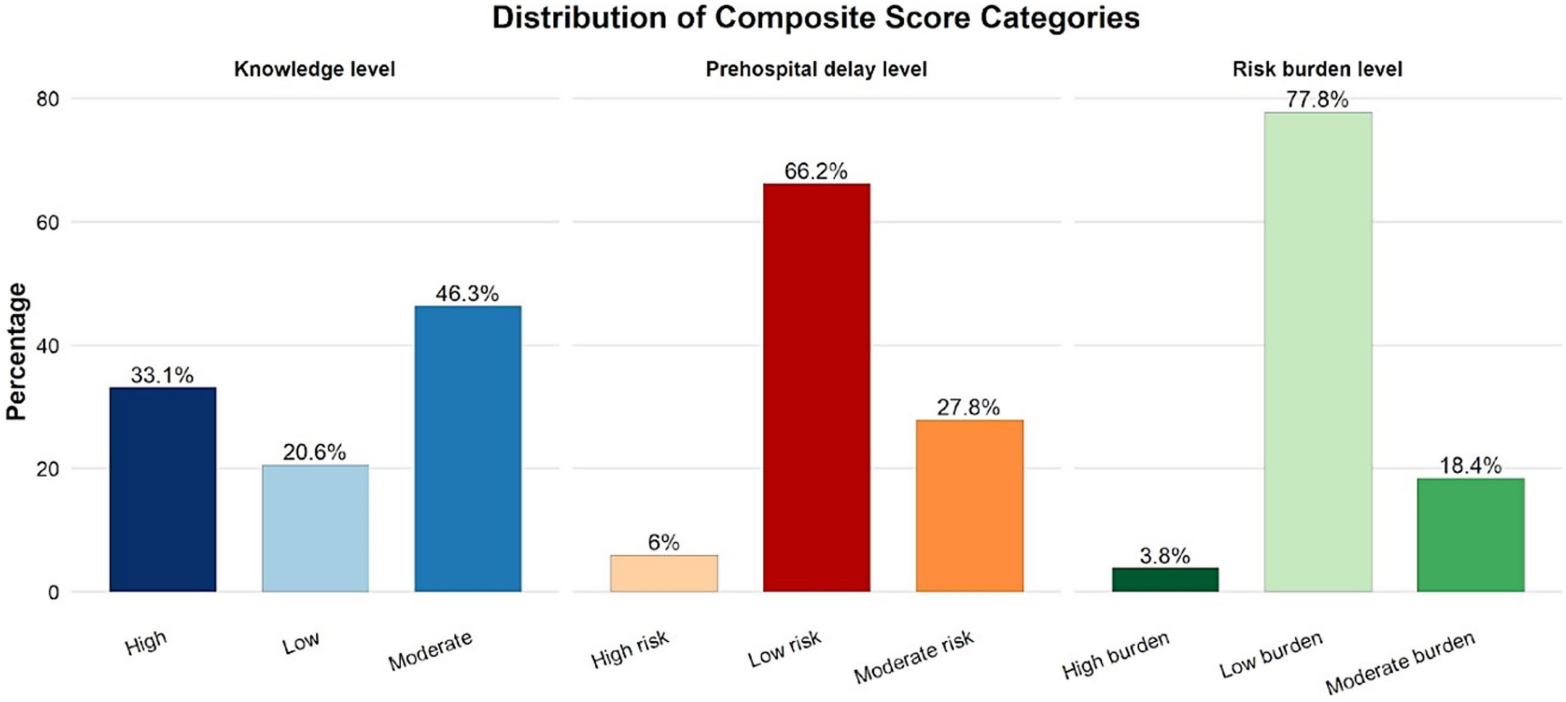
Distribution of composite score categories. Bar charts showing the percentage distribution of participants across three composite measures: knowledge level (low, moderate, high), anticipated prehospital delay risk (high, moderate, low), and cardiovascular risk burden (low, moderate, high). Percentages are calculated within each category group.

When categorized, 20.6% of participants had low knowledge, 46.3% moderate knowledge, and 33.1% high knowledge. For anticipated delay, 6.0% were classified as high delay risk, 27.8% as moderate delay risk, and 66.2% as low delay risk. Most participants (77.8%) had low cardiovascular risk burden (Table 2; Figure 1). The ACS knowledge scale demonstrated acceptable internal consistency (Cronbach’s alpha = 0.77). Internal consistency was not calculated for the anticipated prehospital delay score or cardiovascular risk burden index because these measures were treated as composite indices of heterogeneous response and risk components rather than reflective psychometric scales.

**Table 2:** Distribution of composite score categories.

Variable	Category	*n*	%
Knowledge level	Low knowledge	266	20.6
	Moderate knowledge	599	46.3
	High knowledge	428	33.1
Anticipated prehospital delay level	High delay risk	77	6.0
	Moderate delay risk	360	27.8
	Low delay risk	856	66.2
Risk burden level	Low risk burden	1,006	77.8
	Moderate risk burden	238	18.4
	High risk burden	49	3.8

Compared with national demographic indicators, the recruited online sample was skewed toward groups more likely to participate in social-media surveys. Participants aged 18–29 years represented 56.1% of the sample, while PCBS estimated that youth aged 18–29 years represented about 22% of the total Palestinian population in mid-2023. The sample was also highly educated: 70.0% had a bachelor’s or postgraduate degree, whereas PCBS reported that 18% of Palestinian youth aged 18–29 years had a bachelor’s degree or higher in 2022. Females were also overrepresented in the sample (60.8%). These differences support interpreting the findings as online-sample estimates rather than nationally representative prevalence estimates (16–18).

### Item-level ACS knowledge

Item-level performance showed variability in recognition of ACS symptoms and risk factors (Supplementary Table S1).

Recognition was high for chest pain/pressure (84.2%), shortness of breath (81.9%), and family history (76.9%). Lower recognition was observed for atypical symptoms, including jaw/neck pain (41.5%), nausea/vomiting (53.5%), and back pain (34.0%).

Only 30.3% correctly rejected headache as a typical symptom, and 29.9% identified all major modifiable risk factors. In addition, 60.1% recognized that myocardial infarction can occur without severe chest pain.

### Intended response and knowledge-action gap

A discrepancy was observed between abstract knowledge and intended behavior (Supplementary Table S1).

Although 90.1% identified immediate ambulance activation as the correct action in theory, only 56.2% reported that they would call an ambulance for chest pain lasting more than 5 min. Alternative responses included going by private car (23.1%), waiting for symptom improvement (15.3%), taking analgesics (3.6%), or ignoring symptoms (1.7%).

This discordance was statistically significant (McNemar *χ*^2^ = 381.9, *p* < 0.001), with 40.3% of participants failing to choose the correct action despite knowing it.

The knowledge-action gap was further summarized as discordance between knowing the correct abstract action and not choosing ambulance activation in the chest-pain scenario. Overall, 40.3% of participants failed to choose the correct intended action despite identifying ambulance activation as correct in theory. Additional adjusted modeling of this discordance outcome was not available in the analysis output used for this revision.

### Emergency preparedness and delay barriers

Preparedness for emergency response was limited (Supplementary Table S2).

While 62.8% reported knowing the ambulance number, only 48.6% correctly identified it. Only 39.1% reported no anticipated delay.

The most common barrier was symptom misattribution (52.2%), followed by reluctance to bother others (11.8%), lack of transportation (10.8%), cost concerns (8.9%), and embarrassment (5.9%).

### Bivariate associations

Knowledge score was positively correlated with anticipated prehospital delay score (Spearman’s rho = 0.150, *p* < 0.001), whereas risk burden index was negatively correlated with anticipated prehospital delay score (rho = −0.087, *p* = 0.0017) (Table 3).

**Table 3:** Bivariate associations.

Analysis	Result	*p*-value
Knowledge score vs. prehospital delay score	Spearman’s rho = 0.150	<0.001
Risk burden index vs. prehospital delay score	Spearman’s rho = −0.087	0.0017
Knowledge score by gender	Female: 8.28; Male: 7.33	<0.001
Knowledge score by education	*F* (5,1287) = 18.9	<0.001
Gender vs. prehospital delay category	Chi-square = 15.145, df = 2	0.0005

Female participants had higher knowledge scores than males (8.28 vs. 7.33, *p* < 0.001), and knowledge differed significantly across educational levels. Gender was also associated with anticipated delay category (Table 3).

### Multivariable analyses

In multivariable linear regression, higher ACS knowledge score was independently associated with older age, female sex, higher education, and healthcare worker status (Table 4).

**Table 4:** Multivariable linear regression for knowledge score.

Predictor	Beta coefficient	*p*-value
Age 30–39 years	0.677	0.001
Age 40–49 years	1.179	<0.001
Age 50–59 years	1.388	<0.001
Age ≥ 60 years	0.913	0.083
Male gender	−0.902	<0.001
Preparatory education	1.048	0.262
Bachelor’s degree	2.161	0.011
Secondary education	1.122	0.191
Diploma	1.228	0.162
Postgraduate education	2.524	0.004
Health insurance	0.099	0.541
Healthcare worker	2.336	<0.001

Model fit: Adjusted *R*^2^ = 0.1959; *F* = 27.23; *p* < 0.001.

In a separate model, higher anticipated prehospital delay score was associated with higher knowledge score (*β* = 0.083, *p* < 0.001) and lower risk burden index (*β* = −0.179, *p* < 0.001). Age ≥ 60 years, male sex, and healthcare worker status were also independently associated with anticipated response.

Full regression diagnostic statistics were not available in the analysis output used for this revision. This limits assessment of residual normality, homoscedasticity, multicollinearity, and influential observations and is acknowledged as a limitation.

### Predictors of high delay risk

**Figure 2:**
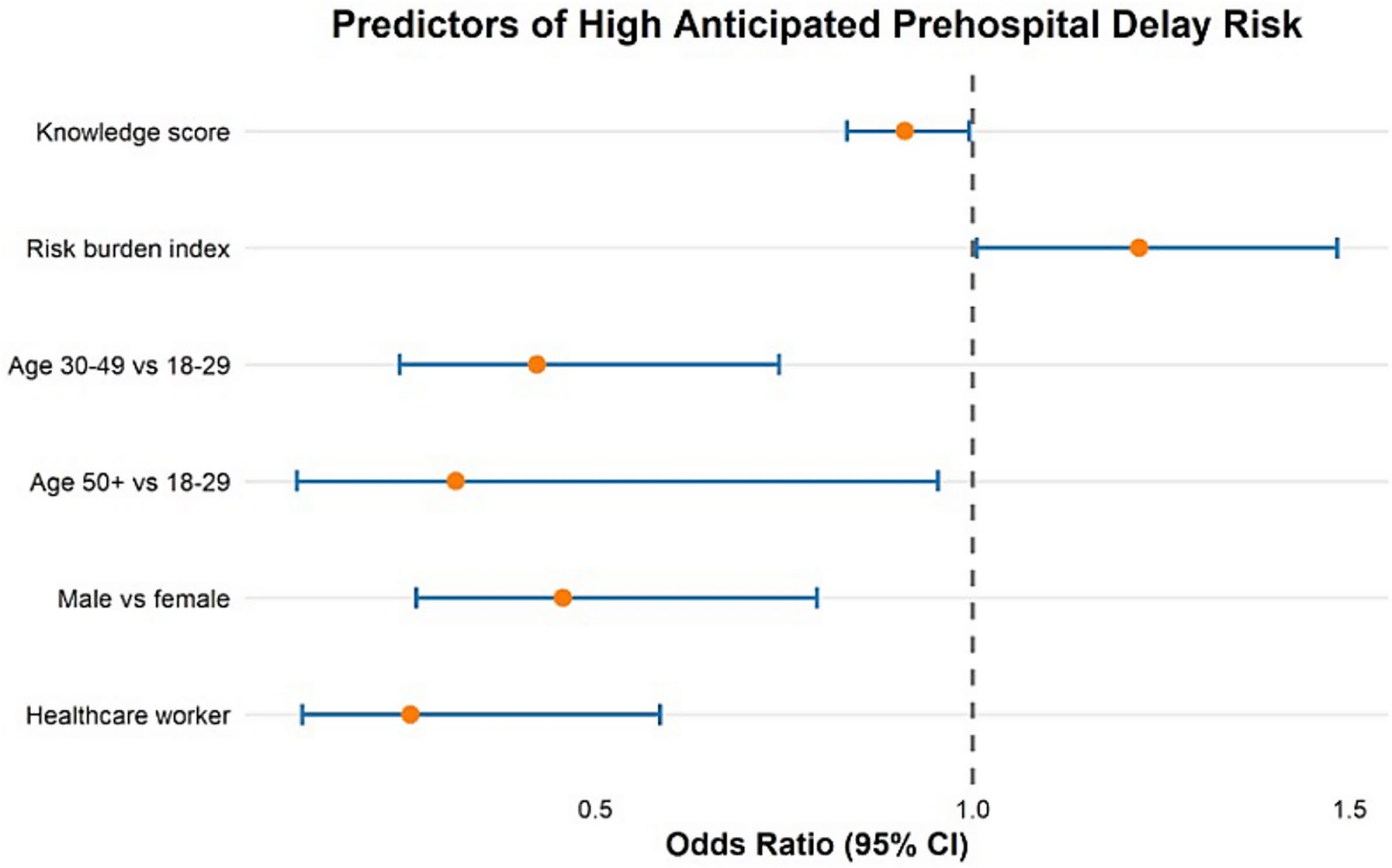
Predictors of high anticipated prehospital delay risk. Forest plot showing odds ratios (OR) and 95% confidence intervals (CI) from logistic regression analysis examining factors associated with high anticipated prehospital delay risk. The dashed vertical line represents the null value (OR = 1).

Binary logistic regression showed that higher knowledge score was associated with lower odds of high anticipated delay risk, whereas higher risk burden index was associated with higher odds (Table 5; Figure 2).

**Table 5:** Binary logistic regression for high anticipated prehospital delay risk.

Predictor	OR	95% CI	*p*-value
Knowledge score	0.91	0.83–1.00	0.047
Risk burden index	1.22	1.01–1.48	0.041
Age 30–49 vs. 18–29	0.42	0.24–0.74	0.003
Age 50 + vs. 18–29	0.32	0.10–0.95	0.047
Male vs. female	0.46	0.26–0.79	0.006
Healthcare worker	0.25	0.11–0.59	0.001

Outcome was coded as 1 = high anticipated prehospital delay risk and 0 = moderate/low delay risk. *P*-values were approximated from the reported odds ratios and 95% confidence intervals where exact *p*-values were not available in the analysis output.

Participants aged ≥ 30 years, males, and healthcare workers had lower odds of high delay risk.

### Cardiovascular risk burden

Cardiovascular risk burden increased with age and was higher in men (Supplementary Tables S5, S6).

No correlation was observed between knowledge score and risk burden (rho = 0.006, *p* = 0.816).

Higher risk burden was associated with lower odds of correct emergency preparedness and greater likelihood of delayed response.

A sensitivity analysis excluding healthcare workers was not available in the analysis output used for this revision. Because healthcare worker status was associated with both knowledge and anticipated response, the absence of this sensitivity analysis should be considered when interpreting the adjusted models.

## Discussion

### Summary of principal findings

This study found moderate ACS knowledge in an online convenience sample of Palestinian adults, with only one-third of participants classified as having high knowledge. Although most participants recognized ambulance activation as the correct response in theory, substantially fewer reported that they would call an ambulance in a specific chest-pain scenario. Higher knowledge was statistically associated with more appropriate anticipated response, but the association was weak, indicating that knowledge alone explains only a small part of intended emergency behavior. Higher cardiovascular risk burden was associated with poorer anticipated response, suggesting that people with greater clinical risk may not necessarily be better prepared to act promptly during suspected ACS.

### Public awareness of ACS symptoms and risk factors

Participants showed uneven ACS knowledge. Classic symptoms such as chest pain and shortness of breath were recognized more frequently than less typical symptoms such as jaw or neck pain, nausea or vomiting, and back pain. This pattern is consistent with a robust body of international evidence showing that public awareness is universally strongest for classic chest-pain presentations and significantly weaker for atypical or less familiar symptom patterns (19). Because atypical symptom recognition is critical for timely help-seeking particularly among female and older adults patient demographics who frequently present without overt chest discomfort, public education in Palestine should emphasize the wider clinical spectrum of ACS presentations rather than chest pain alone (20, 31, 32).

### The knowledge-action gap and prehospital delay

The weak association between knowledge and anticipated response supports the presence of a “knowledge-action gap.” The survey directly measured several intended responses and barriers, including symptom misattribution, uncertainty about urgency, EMS number knowledge, transportation concerns, and reluctance to bother others. Therefore, the findings support a cautious interpretation: information deficits are important, but behavioral and contextual barriers also heavily influence intended emergency response. This critical gap reflects global behavioral models indicating that cognitive awareness does not spontaneously induce protective healthcare-seeking actions during acute ischemic crises (21, 28, 29).

Individuals frequently engage in a cognitive “wait-and-see” approach to evaluate symptom progression or attribute severe symptoms to benign conditions like dyspepsia, inadvertently extending the prehospital window (22, 30). The low adjusted *R*^2^ for the anticipated delay model further indicates that unmeasured psychosocial, access-related, and situational factors likely contribute to this outcome.

Furthermore, when interpreting the linear and logistic regression models utilizing the continuous Anticipated Prehospital Delay Score, substantial caution must be exercised. Although this 0–8 composite index effectively aggregates critical operational components, it fundamentally synthesizes inherently heterogeneous domains including intended action choice, timing thresholds, knowledge of emergency indicators, and perceived behavioral barriers. Because these distinct domains were combined *a priori* as a programmatic index, the assumption of a single underlying construct remains untested. Consequently, the continuous statistical associations identified in our regression analyses should be evaluated as exploratory indicators of multi-faceted behavioral preparedness rather than a standardized, unidimensional psychometric scale.

### Gender disparities: the “knowledge-action” paradox

Gender differences should be interpreted cautiously. Female participants had higher ACS knowledge scores, whereas male participants reported more appropriate anticipated emergency responses and lower odds of high delay risk. Because this survey did not directly measure reasons for gender-specific decision-making, explanations such as symptom appraisal, perceived urgency, caregiving responsibilities, or prior healthcare experiences should be considered hypotheses rather than conclusions from the present data.

However, this paradox mirrors recent epidemiological findings showing that despite superior theoretical literacy, women often face unique psychosocial barriers such as prioritizing familial duties or experiencing clinical validation delays due to atypical presentations that prolong their actual care-seeking intervals (20, 33–35). The finding nonetheless suggests that awareness campaigns should not assume that higher knowledge automatically translates into immediate action and should aggressively address practical, scenario-based decision-making during suspected ACS for both women and men.

### The vulnerability of high-risk populations

A clinically important finding was the association between higher cardiovascular risk burden and poorer anticipated emergency response. This does not prove that high-risk individuals would delay during an actual ACS event, but it suggests a mismatch between objective risk and behavioral readiness. This alarming association aligns with international data demonstrating that patients with chronic clinical conditions like hypertension or diabetes frequently normalize early ischemic signs or mistake them for baseline chronic pathologies (23).

Public health interventions should therefore prioritize individuals with multiple cardiovascular risk factors, and secondary prevention strategies must shift toward hyper-targeted, clinician-led counseling that combines risk-factor management with personalized emergency action planning delivered during routine clinical encounters (24).

Healthcare worker status was associated with higher knowledge and more appropriate anticipated response, indicating that medical familiarity may influence both measured outcomes. Because a sensitivity analysis excluding healthcare workers was not available in the analysis output used for this revision, the influence of healthcare-background participants should be considered when interpreting the main associations.

### Strengths and limitations

- Strengths: The study has several strengths. It included a large sample of 1,293 respondents, assessed both ACS knowledge and anticipated emergency response, and examined cardiovascular risk burden as a predictor of behavioral readiness. The use of item-level results and pre-specified composite scores allowed identification of specific gaps, including limited recognition of atypical symptoms, incomplete EMS preparedness, and discordance between knowing the correct action and intending to perform it. The ACS knowledge scale showed acceptable internal consistency.
- Limitations: Several limitations should be noted. First, the cross-sectional design prevents causal inference. Second, convenience recruitment through social media may have introduced selection bias; the sample overrepresented young adults, women, and highly educated participants and may have underrepresented older adults, individuals with limited internet access, and lower socioeconomic groups. Therefore, the results should not be interpreted as nationally representative prevalence estimates. Third, the study measured self-reported anticipated behavior in hypothetical ACS scenarios rather than actual prehospital delay during a real cardiac event.

Fourth, the newly constructed Anticipated Prehospital Delay Score lacks formal psychometric validation within our specific target population. While the index provides a pragmatic, pre-specified framework to stratify prehospital behavioral risks into low, moderate, and high delay risk thresholds, the assumption of a single underlying construct remains statistically untested. This lack of formal validation should be strongly emphasized when interpreting the regression results based on the continuous score. Future prospective studies employing confirmatory factor analysis (CFA) and structural equation modeling are strongly warranted to evaluate the internal structure, construct validity, and psychometric reliability of this composite score in regional Middle Eastern contexts (36). Furthermore, formal pilot testing, factor analysis, construct validation, regression diagnostics, and a sensitivity analysis excluding healthcare workers were not available in the current analysis output and remain acknowledged limitations that would strengthen future work.

### Public health implications

The findings emphasize the need for public health interventions that go beyond awareness campaigns and incorporate behavior-change strategies, particularly targeting high-risk populations and younger individuals (25). The Palestinian Ministry of Health, healthcare centers, and cardiologists have to conduct a community-based awareness campaign on ACS symptoms recognition and the importance of immediate emergency contact, particularly among young adults and individuals with CVS risk factors. Such interventions should include scenario-based messaging that helps people recognize symptoms and respond without delay.

These findings must be interpreted within the specific Palestinian context, where political instability and ongoing conflict create additional, profound structural barriers to healthcare infrastructure development. Geopolitical factors, transportation restrictions, military checkpoints, limited emergency resources, restricted access to nearby medical facilities, and chronic safety concerns heavily contribute to delayed hospital arrivals, independent of patient psychology (26, 27). Consequently, public health initiatives must be structurally integrated with logistical emergency planning to circumvent these localized infrastructure bottlenecks.

### Implementation and evaluation framework

Future interventions should incorporate structured evaluation frameworks to assess effectiveness, including pre–post knowledge assessment, behavioral outcome tracking, and long-term monitoring of healthcare utilization patterns.

## Conclusion

This study found suboptimal ACS symptom knowledge and a measurable gap between awareness and intended emergency response among Palestinian adults recruited through an online convenience sample. Higher knowledge was associated with more appropriate anticipated response, but the association was weak, and higher cardiovascular risk burden was associated with poorer anticipated response. Because the study assessed hypothetical intended behavior rather than actual prehospital delay, conclusions should be interpreted cautiously. Public health interventions should combine ACS symptom education with practical behavior-change strategies, including scenario-based messaging, EMS activation planning, and targeted outreach to high-risk and lower-literacy groups.

## Data Availability

The raw data supporting the conclusions of this article will be made available by the authors, without undue reservation.
