# Supplementary material for: Public awareness of early symptoms of acute coronary syndrome and its association with anticipated prehospital delay: a cross-sectional study in Palestine
Source: Front Public Health. 2026 Jul 23;14:1856753. doi: 10.3389/fpubh.2026.1856753 (PMC13442815; doi:10.3389/fpubh.2026.1856753)
Supplement: Supplementary file 1 [file Table_1.docx]

**Table S1. Item-level ACS knowledge performance**

| **Item** | **Correct n** | **Correct %** | **95% CI** |
| --- | --- | --- | --- |
| Call ambulance immediately when MI suspected | 1165 | 90.1 | 88.4–91.6 |
| Chest pain/pressure | 1089 | 84.2 | 82.1–86.1 |
| Shortness of breath | 1059 | 81.9 | 79.7–83.9 |
| Family history recognized as a risk factor | 994 | 76.9 | 74.5–79.1 |
| Sudden sweating | 943 | 72.9 | 70.4–75.3 |
| Pain extending to arm/shoulder | 941 | 72.8 | 70.3–75.1 |
| Dizziness/fainting | 813 | 62.9 | 60.2–65.5 |
| Recognized MI can occur without severe chest pain | 777 | 60.1 | 57.4–62.7 |
| Nausea/vomiting | 692 | 53.5 | 50.8–56.2 |
| Jaw/neck pain | 537 | 41.5 | 38.9–44.2 |
| Back pain | 440 | 34.0 | 31.5–36.7 |
| Headache (correctly rejected) | 392 | 30.3 | 27.9–32.9 |
| All four major risk factors identified | 386 | 29.9 | 27.4–32.4 |

**Table S2. Item-level intended response and delay barriers**

| **Item** | **n** | **%** | **95% CI** |
| --- | --- | --- | --- |
| Correct abstract action: call ambulance immediately | 1165 | 90.1 | 88.4–91.6 |
| Would call ambulance immediately for chest pain >5 min | 727 | 56.2 | 53.5–58.9 |
| Would seek help immediately (≤10 min) | 801 | 61.9 | 59.3–64.6 |
| Would seek help within 30 min | 1166 | 90.2 | 88.4–91.7 |
| Would not delay if unsure of symptom severity | 664 | 51.4 | 48.6–54.1 |
| Reported knowing the local ambulance number | 812 | 62.8 | 60.1–65.4 |
| Could state the ambulance number correctly | 628 | 48.6 | 45.9–51.3 |
| Reported no reason that would delay seeking help | 506 | 39.1 | 36.5–41.8 |
| Barrier: pain not considered serious | 675 | 52.2 | 49.5–54.9 |
| Barrier: lack of transportation | 139 | 10.8 | 9.2–12.6 |
| Barrier: reluctance to bother others | 153 | 11.8 | 10.2–13.7 |
| Barrier: cost concern | 115 | 8.9 | 7.5–10.6 |
| Barrier: embarrassment | 76 | 5.9 | 4.7–7.3 |

**Table S3. Selected item-level gender differences**

| **Item** | **Female %** | **Male %** | **p-value** |
| --- | --- | --- | --- |
| Arm/shoulder pain recognition | 78.1 | 64.5 | <0.001 |
| Shortness of breath recognition | 86.5 | 74.8 | <0.001 |
| Sudden sweating recognition | 78.2 | 64.7 | <0.001 |
| Dizziness/fainting recognition | 68.2 | 54.6 | <0.001 |
| Family history recognized as a risk factor | 81.8 | 69.2 | <0.001 |
| Would call ambulance immediately | 52.4 | 62.1 | <0.001 |
| No delay reason reported | 35.0 | 45.6 | <0.001 |

**Table S4. Multivariable logistic regression for immediate ambulance activation in the chest-pain scenario**

| **Term** | **OR (95% CI)** | **p-value** |
| --- | --- | --- |
| Age 30-39 vs 18-29 | 1.03 (0.76–1.39) | 0.837 |
| Age 40-49 vs 18-29 | 1.13 (0.80–1.60) | 0.488 |
| Age 50-59 vs 18-29 | 2.08 (1.22–3.56) | 0.007 |
| Age ≥60 vs 18-29 | 3.43 (1.32–8.88) | 0.011 |
| Male vs female | 1.57 (1.23–2.01) | <0.001 |
| Preparatory vs primary or less | 2.10 (0.50–8.86) | 0.313 |
| Secondary vs primary or less | 2.22 (0.59–8.37) | 0.240 |
| Diploma vs primary or less | 1.62 (0.42–6.31) | 0.485 |
| Bachelor's vs primary or less | 1.49 (0.40–5.57) | 0.554 |
| Postgraduate vs primary or less | 1.34 (0.34–5.22) | 0.673 |
| Health insurance (yes vs no) | 0.89 (0.69–1.13) | 0.336 |
| Healthcare worker (yes vs no) | 1.64 (1.22–2.19) | <0.001 |
| Knowledge score (per 1-point increase) | 1.08 (1.04–1.13) | <0.001 |
| Risk burden index (per 1-point increase) | 0.91 (0.83–1.01) | 0.064 |

Outcome: immediate ambulance activation for chest pain persisting more than 5 minutes. Model pseudo-R² = 0.036.

**Table S5. Multivariable logistic regression for moderate/high cardiovascular risk burden**

| **Term** | **OR (95% CI)** | **p-value** |
| --- | --- | --- |
| Age 30-39 vs 18-29 | 2.56 (1.77–3.72) | <0.001 |
| Age 40-49 vs 18-29 | 4.42 (3.02–6.48) | <0.001 |
| Age 50-59 vs 18-29 | 7.61 (4.50–12.86) | <0.001 |
| Age ≥60 vs 18-29 | 7.10 (3.15–16.02) | <0.001 |
| Male vs female | 1.97 (1.47–2.65) | <0.001 |
| Preparatory vs primary or less | 0.78 (0.17–3.47) | 0.740 |
| Secondary vs primary or less | 0.55 (0.14–2.16) | 0.389 |
| Diploma vs primary or less | 0.32 (0.08–1.36) | 0.123 |
| Bachelor's vs primary or less | 0.33 (0.08–1.30) | 0.113 |
| Postgraduate vs primary or less | 0.32 (0.08–1.30) | 0.110 |
| Health insurance (yes vs no) | 0.89 (0.65–1.21) | 0.440 |
| Healthcare worker (yes vs no) | 1.10 (0.77–1.56) | 0.615 |

Outcome: moderate/high risk burden defined as a burden index ≥3. Model pseudo-R² = 0.110

**Table S6. Age- and sex-specific cardiovascular risk burden gradients**

| **Variable** | **Category** | **Mean risk burden ± SD** | **Moderate/high risk burden n (%)** |
| --- | --- | --- | --- |
| Age group | 18-29 years | 1.45 ± 1.04 | 94 (12.9) |
|  | 30-39 years | 1.78 ± 1.28 | 65 (24.5) |
|  | 40-49 years | 2.32 ± 1.48 | 76 (38.4) |
|  | 50-59 years | 2.71 ± 1.68 | 38 (49.4) |
|  | 60 years and over | 2.63 ± 1.57 | 14 (51.9) |
| Gender | Female | 1.61 ± 1.24 | 140 (17.8) |
|  | Male | 1.97 ± 1.34 | 147 (29.0) |
