# Supplementary material for: Public awareness of early symptoms of acute coronary syndrome and its association with anticipated prehospital delay: a cross-sectional study in Palestine
Source: Front Public Health. 2026 Jul 23;14:1856753. doi: 10.3389/fpubh.2026.1856753 (PMC13442815; doi:10.3389/fpubh.2026.1856753)
Supplement: Supplementary file 2 [file Table_2.DOCX]

**Appendix 1:** Complete Study Questionnaire and Explicit Scoring Algorithms

This appendix provides the exact item wording, coding schema, and scoring algorithms utilized to construct the continuous indices and categorical classifications for the manuscript, ensuring transparency and reproducibility, and allowing for future independent validation.

**Study Questionnaire (Bilingual: English / Arabic Versions)**

Manuscript Title: *Public Awareness of Early Symptoms of Acute Coronary Syndrome and Its Association with Anticipated Prehospital Delay: A Cross-Sectional Study in Palestine*

**Section 1: Information & Informed Consent / الجزء الأول: معلومات الدراسة والموافقة المستنيرة**

Dear Participant,
You are invited to participate in a research project aimed at measuring public awareness of the early symptoms of Acute Coronary Syndrome (Heart Attack) within the Palestinian community, and examining its relationship with the anticipated prehospital delay in seeking medical care. Filling out this questionnaire takes approximately three minutes. Your participation is completely voluntary, and all information collected will remain strictly confidential and used solely for research purposes. Your participation or non-participation will not affect any aspect of your life. Filling out the questionnaire constitutes your implicit consent to participate, provided that you are 18 years of age or older.

عزيزي المشارك / عزيزتي المشاركة،
ندعوك للمشاركة في مشروع بحثي يهدف إلى قياس مستوى الوعي العام بالأعراض المبكرة لمتلازمة الشريان التاجي الحادة (النوبة أو الجلطة القلبية) لدى المجتمع في فلسطين، ودراسة علاقته بالتأخير المتوقع قبل الوصول إلى المستشفى وطلب الرعاية الطبية. علمًا بأن تعبئة الاستبيان تستغرق حوالي ثلاث دقائق فقط. مشاركتك طوعية بالكامل، وجميع المعلومات التي سيتم جمعها ستبقى سرية تمامًا وتُستخدم لأغراض بحثية فقط. كما أن المشاركة أو عدمها لن تؤثر بأي شكل على أي جانب من جوانب حياتك. وتُعدّ تعبئة الاستبيان موافقة ضمنية على المشاركة، على أن يكون عمرك 18 عامًا أو أكثر.

| **English Question / Item** | **النسخة العربية / الفقرة** |
| --- | --- |
| Informed Consent Statement: * ▢ I have read the information provided above and agree to participate in this research questionnaire. | بيان الموافقة المستنيرة: * ▢ لقد قرأت المعلومات الواردة أعلاه ووافقت على المشاركة في هذا الاستبيان البحثي. |
| Participant Code: * [ ___________________________ ] | رمز المشارك (Code): * [ ___________________________ ] |

**Section 2: Demographic Data / الجزء الثاني: البيانات الديموغرافية**

| **English Question / Options** | **النسخة العربية / الخيارات** |
| --- | --- |
| 1. Age (in years): * [ ________ ] | 1. العمر (بالسنوات): * [ ________ ] |
| 2. Gender: * ▢ Male ▢ Female | 2. الجنس: * ▢ ذكر ▢ أنثى |
| 3. Marital Status: * ▢ Single ▢ Married ▢ Divorced / Widowed | 3. الحالة الاجتماعية: * ▢ أعزب / عزباء ▢ متزوج / متزوجة ▢ مطلق/ة أو أرمل/ة |
| 4. Educational Level: * ▢ Less than High School ▢ High School / Diploma ▢ Bachelor's Degree ▢ Postgraduate Degree (Master's / PhD) | 4. المستوى التعليمي: * ▢ أقل من ثانوية عامة ▢ ثانوية عامة / دبلوم ▢ درجة البكالوريوس ▢ دراسات عليا (ماجستير / دكتوراه) |
| 5. Place of Residence (Governorate): * [ ___________________________ ] | 5. مكان السكن (المحافظة): * [ ___________________________ ] |
| 6. Type of Residential Area: * ▢ City ▢ Village ▢ Refugee Camp | 6. نوع منطقة السكن: * ▢ مدينة ▢ قرية ▢ مخيم |
| 7. Do you have health insurance? * ▢ Yes ▢ No | 7. هل لديك تأمين صحي؟ * ▢ نعم ▢ لا |
| 8. Do you work in the healthcare field? * ▢ Yes ▢ No | 8. هل تعمل في المجال الصحي؟ * ▢ نعم ▢ لا |

**Section 3: Knowledge of Acute Coronary Syndrome (ACS) / الجزء الثالث: المعرفة بمتلازمة الشريان التاجي الحادة**

9. Symptom Recognition: Which of the following do you think is a symptom of a heart attack? *
9. التعرف على الأعراض: أي من الآتية تعتقد أنها تُعدّ من أعراض النوبة القلبية؟ *

| **Symptom / العَرَض** | **Yes / نعم** | **No / لا** | **I Don't Know / لا أعرف** |
| --- | --- | --- | --- |
| **Chest pain or pressure** ألم أو ضغط في الصدر | ▢ | ▢ | ▢ |
| **Pain radiating to the arm or shoulder** ألم ممتد إلى الذراع أو الكتف | ▢ | ▢ | ▢ |
| **Pain radiating to the jaw or neck** ألم في الفك أو الرقبة | ▢ | ▢ | ▢ |
| **Shortness of breath** ضيق في التنفس | ▢ | ▢ | ▢ |
| **Nausea or vomiting** غثيان أو تقيؤ | ▢ | ▢ | ▢ |
| **Dizziness or fainting** دوخة أو إغماء | ▢ | ▢ | ▢ |
| **Sudden sweating** تعرق مفاجئ | ▢ | ▢ | ▢ |
| **Back pain** ألم في الظهر | ▢ | ▢ | ▢ |
| **Headache** صداع | ▢ | ▢ | ▢ |

| **English Question & Options** | **النسخة العربية والخيارات** |
| --- | --- |
| 10. Which of the following is considered a risk factor for a heart attack? (Select all that apply) * ▢ Smoking ▢ Hypertension (High blood pressure) ▢ Diabetes ▢ High cholesterol ▢ Obesity / Unhealthy diet ▢ Physical inactivity ▢ Advanced age ▢ Psychological stress | 10. أي من التالي يُعتبر عامل خطورة للجلطة القلبية؟ (يمكنك اختيار أكثر من إجابة) * ▢ التدخين ▢ ارتفاع ضغط الدم ▢ السكري ▢ ارتفاع الكوليسترول ▢ السمنة / النظام الغذائي غير الصحي ▢ الخمول البدني / قلة الحركة ▢ التقدم في العمر ▢ الضغوطات النفسية |
| 11. Does a family history of heart disease increase the risk? * ▢ Yes ▢ No ▢ I don't know | 11. هل التاريخ العائلي لأمراض القلب يزيد من خطر الإصابة؟ * ▢ نعم ▢ لا ▢ لا أعرف |
| 12. What is the most appropriate action when a heart attack is suspected? * ▢ Call the local ambulance immediately ▢ Drive yourself or have someone drive you to the hospital in a private car ▢ Wait and see if symptoms improve ▢ Take a painkiller or traditional herbal remedy | 12. ما هو الإجراء الأنسب عند الاشتباه بجلطة قلبية؟ * ▢ الاتصال بالإسعاف المحلي فوراً ▢ التوجه للمستشفى بالسيارة الخاصة فوراً ▢ الانتظار لرؤية ما إذا كانت الأعراض ستتحسن ▢ تناول مسكن آلام أو أعشاب تقليدية |
| 13. Can a heart attack occur without severe chest pain? * ▢ Yes ▢ No ▢ I don't know | 13. هل يمكن أن تحدث الجلطة القلبية دون ألم شديد في الصدر؟ * ▢ نعم ▢ لا ▢ لا أعرف |

**Section 4: Anticipated Behavior Upon Symptom Onset / الجزء الرابع: السلوك المتوقع عند حدوث أعراض**

| **English Question & Options** | **النسخة العربية والخيارات** |
| --- | --- |
| 14. If you experience sudden chest pain that lasts for more than 5 minutes, what would you do first? * ▢ Call the national ambulance service ▢ Go to the nearest emergency department immediately ▢ Contact a family member or friend for advice ▢ Rest and wait for the pain to pass ▢ Take self-medication (e.g., aspirin, antacids) | 14. إذا شعرت بألم مفاجئ في الصدر يستمر أكثر من 5 دقائق، ماذا ستفعل أولاً؟ * ▢ الاتصال بالإسعاف الوطني ▢ التوجه لأقرب قسم طوارئ فوراً ▢ الاتصال بأحد أفراد العائلة أو الأصدقاء للاستشارة ▢ الراحة والانتظار ليزول الألم تلقائياً ▢ أخذ دواء ذاتي (مثل الأسبرين أو مضاد حموضة) |
| 15. How long would you wait before seeking medical help? * ▢ Immediately (Within less than 15 minutes) ▢ 15–30 minutes ▢ 31–60 minutes ▢ More than 1 hour ▢ More than 2 hours / Until the next day | 15. كم من الوقت ستنتظر قبل طلب المساعدة الطبية؟ * ▢ فوراً (أقل من 15 دقيقة) ▢ 15–30 دقيقة ▢ 31–60 دقيقة ▢ أكثر من ساعة ▢ أكثر من ساعتين / لليوم التالي |
| 16. If you are not sure about the severity of the symptoms, would you delay seeking help? * ▢ Yes ▢ No | 16. إذا لم تكن متأكداً من خطورة الأعراض، هل ستؤخر طلب المساعدة؟ * ▢ نعم ▢ لا |
| 17. Do you know the local ambulance number? * ▢ Yes ▢ No | 17. هل تعرف رقم الإسعاف المحلي؟ * ▢ نعم ▢ لا |
| 18. If yes, what is the local ambulance number? [ ___________________________ ] | 18. إذا كنت تعرف رقم الإسعاف المحلي، فما هو الرقم؟ [ ___________________________ ] |
| 19. What reasons might make you delay seeking medical help? (Select all that apply) * ▢ Hoping the symptoms will go away on their own ▢ Fear of being hospitalized or hearing bad news ▢ Fear of the financial costs ▢ Not wanting to bother family members or medical staff ▢ Lack of available transportation ▢ Attributing the symptoms to other causes (e.g., gastric reflux, muscle strain) ▢ Lack of escort / caregiver | 19. ما الأسباب التي قد تجعلك تؤخر طلب المساعدة؟ (يمكن اختيار أكثر من إجابة) * ▢ الأمل في أن تزول الأعراض من تلقاء نفسها ▢ الخوف من دخول المستشفى أو سماع أخبار سيئة ▢ الخوف من التكاليف المادية ▢ عدم الرغبة في إزعاج أفراد العائلة أو الطاقم الطبي ▢ عدم توفر وسائل النقل ▢ إرجاع الأعراض لأسباب أخرى (مثل ارتداد مريئي، شد عضلي) ▢ عدم وجود مرافق |

**Section 5: Cardiovascular Risk Factors / الجزء الخامس: عوامل الخطورة القلبية**

| **English Question & Options** | **النسخة العربية والخيارات** |
| --- | --- |
| 20. Do you currently smoke? * ▢ Yes ▢ No | 20. هل تدخن حالياً؟ * ▢ نعم ▢ لا |
| 21. If yes: number of cigarettes daily: [ ________ ] | 21. إذا نعم: عدد السجائر يومياً: [ ________ ] |
| 22. Have you been diagnosed with hypertension (high blood pressure)? * ▢ Yes ▢ No ▢ I don't know | 22. هل تم تشخيصك بارتفاع ضغط الدم؟ * ▢ نعم ▢ لا ▢ لا أعرف |
| 23. Have you been diagnosed with diabetes? * ▢ Yes ▢ No ▢ I don't know | 23. هل تم تشخيصك بالسكري؟ * ▢ نعم ▢ لا ▢ لا أعرف |
| 24. Have you been diagnosed with high cholesterol? * ▢ Yes ▢ No ▢ I don't know | 24. هل تم تشخيصك بارتفاع الكوليسترول؟ * ▢ نعم ▢ لا ▢ لا أعرف |
| 25. Is there a family history of cardiovascular disease? * ▢ Yes ▢ No ▢ I don't know | 25. هل يوجد تاريخ عائلي لأمراض القلب؟ * ▢ نعم ▢ لا ▢ لا أعرف |
| 26. Do you engage in physical activity for ≥150 minutes per week? * ▢ Yes ▢ No | 26. هل تمارس نشاطاً بدنياً لمدة ≥150 دقيقة أسبوعياً؟ * ▢ نعم ▢ لا |
| 27. Do you consume fruits and vegetables daily? * ▢ Yes ▢ No | 27. هل تتناول الفواكه والخضروات يومياً؟ * ▢ نعم ▢ لا |

**Thank you very much for your time and cooperation! 😊
شكرًا جزيلًا على وقتك وتعاونك! 😊**

1. ACUTE CORONARY SYNDROME (ACS) KNOWLEDGE SCORE

======================================================================

* Scope: Section II – Evaluates baseline public literacy regarding symptom recognition, risk factors, and core action protocols.

* Scoring Rule: Dichotomous coding where each correct answer = 1 point, and any incorrect or "Don't know" response = 0 point (Cumulative Range: 0–13 points).

Item-Level Operational Wording & Coding:

* Symptom Recognition (9 Items): "Do you believe the following manifestations can be an early sign of a heart attack (ACS)?"

1. Chest pain, pressure, or tightness (Correct: Yes = 1, No/Don't know = 0)

2. Pain radiating to the left arm or shoulder (Correct: Yes = 1, No/Don't know = 0)

3. Neck, jaw, or throat pain (Correct: Yes = 1, No/Don't know = 0)

4. Shortness of breath / dyspnea (Correct: Yes = 1, No/Don't know = 0)

5. Sudden diaphoresis / heavy sweating (Correct: Yes = 1, No/Don't know = 0)

6. Nausea or vomiting (Correct: Yes = 1, No/Don't know = 0)

7. Dizziness, lightheadedness, or fainting (Correct: Yes = 1, No/Don't know = 0)

8. Back pain (Correct: Yes = 1, No/Don't know = 0)

9. Epigastric pain / heartburn / indigestion (Correct: Yes = 1, No/Don't know = 0)

* Cardiovascular Risk Factors (2 Items): "Identify if the following options significantly elevate an individual's objective risk of developing a heart attack."

10. Chronic clinical conditions (e.g., Hypertension, Diabetes, Dyslipidemia) (Correct: Yes = 1)

11. Unhealthy lifestyle modifications (e.g., Smoking, physical inactivity, poor diet) (Correct: Yes = 1)

* Action Knowledge (2 Items): "Identify the appropriate urgent actions mandated when an acute cardiac crisis is suspected."

12. Immediate recognition of clinical urgency without attempting self-medication remedies (Correct: Yes = 1)

13. Recognizing that emergency medical transport is superior to delayed private transport (Correct: Yes = 1)

Categorical Stratification Thresholds:

* Low Knowledge: Continuous score of 0–5 points

* Moderate Knowledge: Continuous score of 6–9 points

* High Knowledge: Continuous score of 10–13 points

======================================================================

2. ANTICIPATED PREHOSPITAL DELAY SCORE

======================================================================

* Scope: Section III – Synthesizes expected behavioral trajectories, temporal intent, and basic emergency literacy when encountering acute ACS symptoms.

* Scoring Rule: Point allocation varies dynamically across 4 distinct behavioral dimensions to yield a cumulative aggregate index (Cumulative Range: 0–8 points).

Item-Level Operational Wording & Coding:

1. Initial Action Choice:

* Wording: "If you or a person next to you experiences sudden, severe, crushing chest pain, what is the very first action you intend to take?"

* Coding Matrix:

- Call local Emergency Medical Services (EMS) / Ambulance immediately = 3 points

- Go to the hospital emergency department directly (via private vehicle/taxi) = 2 points

- Wait and see if the symptoms resolve spontaneously = 1 point

- Take an over-the-counter painkiller / ignore the symptoms = 0 points

2. Anticipated Time Interval Until Seeking Medical Help:

* Wording: "Within what timeframe from symptom onset would you consider it critical to reach a medical facility?"

* Coding Matrix:

- Immediately (<= 10 minutes) = 3 points

- 10–30 minutes = 2 points

- 30–60 minutes = 1 point

- More than 1 hour / Delayed = 0 points

3. Behavioral Delay if Symptom Origin is Unsure / Atypical:

* Wording: "Would you hesitate or delay seeking immediate medical care if the symptoms were atypical or you were unsure if it was a true heart attack?"

* Coding Matrix:

- No (Would not delay care) = 1 point

- Yes (Would delay care) = 0 points

4. Emergency Literacy (Knowledge of Local EMS/Ambulance Number):

* Wording: "Do you know the exact direct telephone emergency number to contact the national ambulance services in Palestine?"

* Coding Matrix:

- Yes (Correctly specifies the national emergency number) = 1 point

- No / Incorrect response = 0 points

Composite Risk Stratification Thresholds:

* High Delay Risk (Unfavorable Behavior): Cumulative score of 0–2 points

* Moderate Delay Risk: Cumulative score of 3–5 points

* Low Delay Risk (Favorable Behavior / Good Preparedness): Cumulative score of 6–8 points

======================================================================

3. CARDIOVASCULAR RISK BURDEN INDEX

======================================================================

* Scope: Section IV – Quantifies individual cumulative clinical and lifestyle risk parameters present within the participant.

* Scoring Rule: 1 Point is allocated per confirmed risk factor present based on dichotomous (Yes/No) self-reported mapping (Cumulative Range: 0–7 points).

Operational Definitions for Risk Factor Items:

1. Current Smoking Status: Active smoking of traditional tobacco products, shisha, or electronic cigarettes (Yes = 1, No = 0).

2. Hypertension: Prior formal clinical diagnosis by a physician or current use of anti-hypertensive medication (Yes = 1, No = 0).

3. Diabetes Mellitus: Prior formal clinical diagnosis of Type 1 or Type 2 Diabetes or current regular use of insulin/oral hypoglycemic agents (Yes = 1, No = 0).

4. Dyslipidemia: Prior documented clinical history of high cholesterol/triglycerides or use of lipid-lowering therapies (Yes = 1, No = 0).

5. Family History of Premature CVD: Confirmed biological first-degree relative (parent or sibling) diagnosed with premature ischemic heart disease (Male < 55 years; Female < 65 years) (Yes = 1, No = 0).

6. Physical Inactivity: Failing to accumulate at least 150 minutes of moderate-intensity, or 75 minutes of vigorous-intensity aerobic physical activity throughout an average week (Yes = 1, No = 0).

7. Poor Diet: Not consuming fresh fruits or vegetables on a daily baseline (Yes = 1, No = 0).

Categorical Stratification Thresholds:

* Low Risk Burden: Continuous score of 0–2 points

* Moderate Risk Burden: Continuous score of 3–4 points

* High Risk Burden: Continuous score of 5–7 points
